# Associations Among Health Literacy, Decision Self-Efficacy, and Decisional Conflict in Stroke Survivor–Caregiver Dyads

**DOI:** 10.1097/jnr.0000000000000754

**Published:** 2026-06-29

**Authors:** Ya-Ting LIU, Si-Xun ZHANG, Mei-Sin CHONG, Ying-Chen DUAN, Yi-Nan SHI, Zhen-Xiang ZHANG, Wen-Na WANG, Xin LI, Bei-Lei LIN, Nuo SHI, Kwan Ching WONG, Yong-Xia MEI

**Affiliations:** 1 School of Nursing and Health, Zhengzhou University, Zhengzhou, China; 2 Alice Lee Centre for Nursing Studies, Yong Loo Lin School of Medicine, National University of Singapore, Republic of Singapore; 3 The Second Affiliated Hospital of Zhengzhou University, China; 4 School of Nursing, The Hong Kong Polytechnic University, SAR, China

**Keywords:** actor–partner interdependence mediation model, health literacy, decision self-efficacy, decisional conflict, stroke

## Abstract

**Background:**

The cognitive and physical impairments often experienced by stroke survivors make health care decisions challenging for both survivors and their caregivers. Although poststroke care decision-making involves complex factors such as health literacy and decision self-efficacy, research on the interactions among health literacy, decision self-efficacy, and decisional conflict within stroke survivor–caregiver dyads remains limited.

**Purpose:**

This study was designed to explore the inter-relationships among health literacy, decision self-efficacy, and decisional conflict in stroke survivor–caregiver dyads.

**Methods:**

This cross-sectional study, conducted from September 2023 to April 2024, included 305 pairs of Chinese stroke survivor–caregiver dyads. The All Aspects of Health Literacy Scale, Decision Self-Efficacy Scale, and Decisional Conflict Scale were used to collect data from both stroke survivors and their caregivers. A dyadic analysis was conducted using the actor–partner interdependent mediation model.

**Results:**

In terms of actor effects, decision self-efficacy was found to respectively mediate health literacy and decisional conflict in stroke survivors (β = −0.511, *p* < .001) and their caregivers (β = 0.212, *p* = .006). In terms of the partner effect, caregiver decision self-efficacy was found to relate negatively to survivor decisional conflict and to mediate the relationship between caregiver health literacy and survivor decisional conflict (β = −0.236, *p* = .019).

**Conclusions:**

The results of this study indicate that, in stroke survivors, higher health literacy has a direct reducing effect on perceived decisional conflict. In light of this, dyadic-based intervention strategies should be developed. Examples include joint workshops designed to improve stroke survivor–caregiver shared information interpretation and comprehension, and the development of dyadic-oriented decision aids to help both dyad partners achieve information symmetry and consensus in their health care decision-making.

## Introduction

Stroke is the second leading cause of death and a main cause of long-term disability worldwide (Martin et al., 2024). With an aging global population, stroke is becoming an increasingly significant health concern. Between 1990 and 2019, the absolute incidence and prevalence of stroke rose, respectively, by 70% and 85% worldwide (Hilkens et al., 2024). After stroke, survivors often face both physical and cognitive dysfunctions, with studies showing that approximately half of all stroke survivors become disabled and that one-third rely on others for assistance with activities of daily living (Pu et al., 2023). In China, collective cultural mores emphasize the importance of family responsibility and cohesion. Therefore, most patients choose home care during rehabilitation, with related tasks usually performed by the patient’s family members, placing a heavy caregiving burden on these caregivers.

The impact of stroke goes beyond the individual patient to affect caregivers, who bear important support and care responsibilities in the realms of daily life care, medical decision-making, and rehabilitation management (Andrades-González & Molina-Mula, 2022). In China, where collectivist cultural mores are prevalent, family caregivers play a central role in patient health decision-making, with Chinese family caregivers often acting as “co-decision-makers” in health care decision-making and significantly influencing final treatment choices (Mei et al., 2023). However, the long-term and complex burden of caregiving often leads to psychological distress and decision-making fatigue, which in turn affects the patient's recovery process and the quality of medical decision-making (Bartoli et al., 2024).

In the context of home rehabilitation care for stroke survivors, survivors and caregivers are highly susceptible to decisional conflicts due to limited understanding of treatment options, insufficient information, and/or inconsistent values (Mei et al., 2023). Decisional conflict refers to the hesitation and uneasiness of individuals in making health care decisions due to insufficient information, difficulty in clarifying values, and/or lack of support systems (Pel-Littel et al., 2024). Decisional conflict not only delays the adoption of necessary medical behaviors but also may reduce treatment compliance, increase psychological burden, and thus affect rehabilitation outcomes (Goh et al., 2022). However, the focus of most studies in the literature is on investigating decisional conflict in single subjects. Few have been designed to explore the decision-making dynamics of stroke survivor–caregiver dyads and whether health literacy and decision self-efficacy affect intradyad decisional conflict.

### Background

Stroke survivors face significantly elevated risks of morbidity, disability, mortality, recurrence, and economic burden and, after treatment, frequently require long-term rehabilitation and experience a range of sequelae with differing degrees of severity (Hilkens et al., 2024). Rehabilitation care decision-making often requires comprehensive consideration of disease characteristics, changes in personal health status, family relationship dynamics, economic conditions, and other factors, making the decision-making process full of complexity and uncertainty (Guo et al., 2024). Decisional conflict in patients has been shown to be strongly associated with poor recovery outcomes and potentially associated with higher negative emotions and increased risk of decision regret (Aoki & Nakayama, 2023).

In addition, caregivers of stroke survivors face complex decision-making challenges in terms of medical treatment options, rehabilitation, lifestyle modifications, and medication use. Also, they face uncertainty regarding the risks and benefits of decisions and are more likely to experience anxiety and stress, which can affect the quality of their decisions (Fadem, 2023). Furthermore, caregivers must cope with the emotional loss of the patient (Glick et al., 2018) and balance the interests of the entire family. This increased psychological distress and responsibility may further exacerbate decisional conflict (Marziliano et al., 2023), which may ultimately impede the patient's recovery process. The findings of a qualitative study indicate that the perceptions of patients and their surrogate decision-makers may diverge significantly during the decision-making process (Zhang et al., 2021). However, as most studies in the literature focus on either the patient or caregiver perspective, there is an urgent need to explore the decisional conflicts of stroke survivors and their caregivers from a dichotomous perspective.

Health literacy, which refers to an individual’s ability to access, understand, evaluate, and effectively use health information, is widely recognized as a key factor influencing disease management and health decision-making (Muscat et al., 2021). The concept of health literacy is particularly important because it relates not only to individual health but also to sociocultural, economic, and educational factors (Berkman et al., 2011). Patients with lower health literacy often struggle to fully understand their condition (Goh et al., 2022), leading to delayed diagnosis. Patients with low health literacy and poorer health status have been shown to experience more decisional conflict (van Lent et al., 2022). Also, the results of a study of older adults support the idea that patient health literacy significantly and negatively relates to decisional conflict (Pel-Littel et al., 2024). Furthermore, health literacy in stroke survivor caregivers must be better elucidated, as these individuals not only assist patients with medications, daily management, and caregiving but also influence related health care and treatment decisions (Fadem, 2023). Low health literacy among caregivers can affect appropriate care and, therefore, patient health outcomes (Yuen et al., 2023). In practice, caregivers often assume caregiving roles with little or no prior preparation. In addition, most studies on caregiver health literacy have focused on specific groups, for example, cancer patients (Yuen et al., 2023) and parents of children (Lynn et al., 2020). The relative lack of research on health literacy among caregivers of stroke survivors makes it difficult to comprehensively assess its impact on stroke survivor recovery outcomes.

Bandura’s theory of self-efficacy emphasizes the influence of an individual’s assessment of their own abilities and beliefs on behavioral choices and levels of effort (Bandura, 1977). Decision self-efficacy refers to confidence in one’s decisional ability. The level of information provided to an individual relates positively to their confidence in making decisions about their own health and to reducing decisional conflict (Lee & Bryant-Lukosius, 2023). In stroke survivor caregivers, decision self-efficacy reflects their confidence in assisting the patient in making health care decisions, with higher self-efficacy associated with greater confidence in decision-making, lower confusion and anxiety, and better quality of care provided (Roshan et al., 2023). Therefore, improving decision self-efficacy is essential for stroke survivor–caregiver dyads to effectively manage complex medical, rehabilitation, and other decisions.

Interdependence theory emphasizes the emotional and behavioral interactions between individuals in intimate relationships in which the participants mutually influence each other (Wickham & Knee, 2012). Under this theory, individuals do not exist in isolation from each other’s emotional experiences and behavioral responses when confronted with stressors, but rather interact to form dynamically changing relational patterns. In health care decision-making situations, patients and caregivers face disease challenges together, and their decision-making behaviors are intertwined (Wickham & Knee, 2012). For example, the health literacy level of caregivers affects the quality of the decision support they provide to patients, which in turn affects patients’ decision self-efficacy and the level of decisional conflict (Yuen et al., 2023). This suggests the existence of a bidirectional transmission process between patients and caregivers. Interdependence theory provides an important framework for understanding the interactions among decisional conflict, health literacy, and decision self-efficacy in the patient–caregiver dyadic and supports the development of intervention strategies based on dyadic interactions.

**Figure 1:**
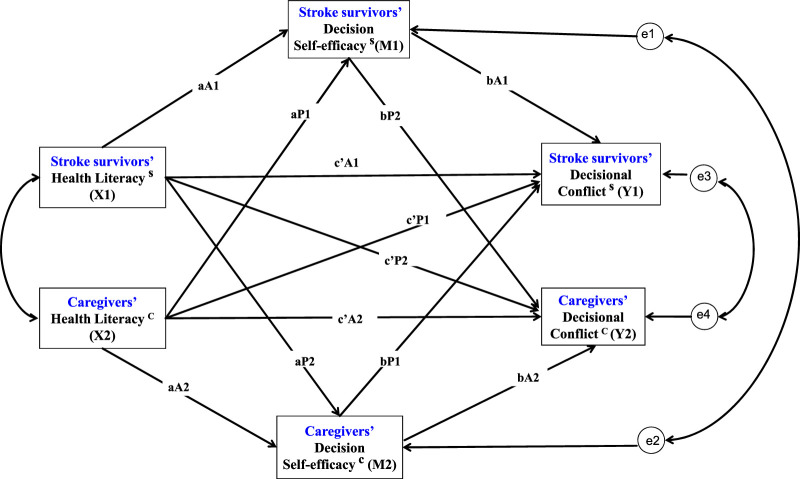
The Hypothetical Model of This Study *Note.* a = direct effect of health literacy on decision self-efficacy; b = direct effect of decision self-efficacy on decisional conflict; c’ = direct effect of health literacy on decisional conflict; A = actor effect; P = partner effect. ^S^ Stroke survivors. ^C^ Caregiver.

The following hypotheses are investigated in this study, see Figure 1.

Hypothesis 1: In individuals, health literacy relates negatively both to their internal level of decisional conflict and to the level of decisional conflict with their partner.

Hypothesis 2: In individuals, decision self-efficacy relates negatively both to their internal level of decisional conflict and to the level of decisional conflict with their partner.

Hypothesis 3: In individuals, health literacy relates positively to their own and their partners’ levels of self-efficacy in decision-making.

Hypothesis 4: In individuals, decision self-efficacy mediates the relationship between their partner’s health literacy as well as between their own and their partner’s decisional conflict.

## Methods

### Design

This cross-sectional descriptive study was conducted from September 2023 to April 2024. The Strengthening the Reporting of Observational Studies in Epidemiology (STROBE) guidelines were applied.

### Participants

Stroke survivor–caregiver dyads were recruited from the neurology and rehabilitation departments of six tertiary hospitals in Henan Province. The inclusion criteria for stroke survivors were (a) stroke survivors diagnosed using computed tomography (CT) or magnetic resonance imaging (MRI), (b) age ≥ 18 years, (c) normal language (Token test score ≥ 17) and cognitive (Mini-Mental State Examination score ≥ 17) functions, and (d) providing informed consent and volunteering to participate. The inclusion criteria for caregivers were (a) designated by the stroke survivor as their primary caregiver, (b) providing informal care to the patient without remuneration, (c) age ≥ 18 years, and (d) providing informed consent and volunteering to participate. The exclusion criteria for both participants were (a) suffering from a (concomitant) serious chronic condition and (b) currently participating in another psychological intervention program.

Based on the statistical efficacy requirements of the Actor–Partner Interdependence Mediation Model (APIMeM), Ledermann and Macho's (2009) sample size calculation method for distinguishable dyadic mediation models was used. Based on the hypothesized path effect, the actor effect was set at *f*² = 0.14, the partner effect at *f*² = 0.11, and the mediated path effect at *f*² = 0.21, with the significance level of the two-sided test set as α = .05 and the efficacy of the statistical test set as (1-β) = 0.80. The result indicated the minimum sample size required for model fitting to be 285 pairs. Considering an invalid rate of 10%, a recruitment target of 314 pairs was set. Data from 305 valid sample pairs were included in the analysis, which meets the statistical efficacy of the APIMeM analysis.

### Data Collection

Study data were collected using a questionnaire. A uniform guideline was used to explain the questionnaire, and both stroke survivors and their caregivers were required to provide written informed consent to be included. Survivors and caregivers were surveyed separately to avoid interference bias. Data entry was performed independently by two researchers, with any discrepancies resolved by a third researcher. Three hundred fifteen dyadic pairs agreed to participate in this study, and after verifying the completed questionnaires for completeness, 305 stroke survivor–caregiver dyadic pairs were included in the analysis (valid recovery rate: 96.83%).

### Measurement

#### Sociodemographic and Clinical Characteristics

The sociodemographic data collected from all participants included age, gender, educational level, and knowledge of the disease. The clinical characteristics data collected from the stroke survivors included the number of strokes and the type of stroke.

#### All Aspects of Health Literacy Scale (AAHLS)

The All Aspects of Health Literacy Scale, originally developed by Chinn and McCarthy in 2013, was designed to assess functional, communicative, and critical health literacy (Chinn & McCarthy, 2013). The scale consists of 3 dimensions and 11 entries, which are scored on a 3-point Likert scale. The total potential score range is 11–33, and higher scores indicate higher levels of individual health literacy. In 2017, Wu et al. translated the scale into Chinese and validated its reliability on a Chinese population with an overall Cronbach’s alpha coefficient of .811 (Wu et al., 2017). In this study, the Cronbach’s alpha was .838 for stroke survivors and .845 for caregivers.

#### Decision Self-Efficacy (DSE) Scale

The DSE scale, developed by Bunn and O’Connor in 1996, has mainly been used to assess confidence in making informed health care–related choices (Bunn & O’Connor, 1996). This scale consists of 11 items and is scored on a 5-point Likert scale. The total score is calculated by adding the average of all item scores and multiplying the result by 25, giving a total possible score range of 0–100. Higher scores indicate a higher level of decision-making self-efficacy.

Wang et al. translated the scale into Chinese in 2021 and validated it on a group of primary liver cancer patients, resulting in a Cronbach’s alpha coefficient for the scale of .918 (Wang et al., 2021). In this study, the Cronbach’s alpha was .934 for stroke survivors and .902 for caregivers.

#### Decisional Conflict Scale (DCS)

The DCS was developed by O’Connor (1995) to assess the level of uncertainty regarding an action to be taken. The scale consists of 5 dimensions and 16 items, and is scored on a 5-point Likert scale. The final scale score is calculated as the average of the 16 items multiplied by 25, with a total possible score range of 0–100. Scale scores <25 indicate no decisional conflict; 25–37.5 indicate moderate decisional conflict; and >37.5 indicate high decisional conflict, with higher scores indicative of greater decisional conflict. Wang et al. first applied the scale in China in 2019, showing good internal consistency in a population of rectal cancer patients with a Cronbach’s alpha coefficient of .886 (Wang et al., 2019). In this study, the Cronbach’s alpha was .892 for stroke survivors and .857 for caregivers.

### Ethical Considerations

This study was reviewed and approved by the Zhengzhou University Life Sciences Ethics Review Board (ZZUIRB2020-053). All of the participants were informed of the study's purpose and process. Both stroke patients and caregivers were required to provide informed consent to complete and submit the survey.

### Data Analysis

In this study, IBM SPSS Statistics 26.0 (IBM Corp., Armonk, NY, USA) was used to statistically describe general information in terms of means, standard deviations, frequencies, and percentages. The correlations between dyadic variables were analyzed using R (Version 4.5.1; R Core Team) on the corrplot package (Version 0.92), and a correlation heat map was plotted. Mplus 8.3 (Muthén & Muthén, Los Angeles, CA, USA) was used to conduct the APIMeM analysis.

Given the interdependence of the dual data, APIMeM was employed in data analysis (Ledermann & Macho, 2009). The actor effect is defined as the effect of one person’s independent variable on their own dependent variable, while the partner effect is defined as the effect of one person’s independent variable on their partner’s dependent variable. In this study, APIMeM was utilized to examine the effects of health literacy on decisional conflict in stroke survivor–caregiver dyads and the moderating effect of decision self-efficacy. Path a shows the influence of health literacy (X) on decision self-efficacy (M), path b shows the influence of decision self-efficacy (M) on decisional conflict (Y), and path c’ shows the influence of health literacy (X) on decisional conflict (Y). The actor effect for patients is the effect from X1 to Y1, and the partner effect for patients is the effect from X2 to Y1. The caregiver’s actor effect is the effect from X2 to Y2, and the caregiver’s partner effect is the effect from X1 to Y2. The research hypothesis model for this study is illustrated in Figure 1.

Modeling using structural equation modeling and maximum likelihood estimation was assessed using skewness and kurtosis metrics, with variables having an absolute value of skewness < 2 and an absolute value of kurtosis <7, which meets the assumption of normality. Model fit was evaluated using χ^2^, degree of freedom (*df*), root mean square error of approximation (RMSEA), goodness of fit index (GFI), incremental fit index (IFI), comparative fit index (CFI), and Tucker-Lewis index (TLI; Raykov et al., 1991). The resulting respective values of χ^2^/*df* < 5, RMSEA ≤ 0.08, GFI ≥ .90, IFI ≥ .90, and TLI ≥ 0.90 indicate good model fit (Comulada, 2021). The mediation effects were tested using the bias-corrected percentile bootstrap method with 5,000 resamples.

## Results

### Dyad Demographic Characteristics

In the 305 stroke survivor–caregiver dyads (Table 1), the mean age of the patients was 58.42 years (*SD* = 11.92). Two-thirds (66.3%) of the patients had suffered ischemic stroke, and a majority (66.9%) were male. The mean age of the caregivers was 46.08 years (*SD* = 14.36). In terms of relationship to the patient, 52.8% the caregivers were their children, and 39.7% were their spouse.

**Table 1:** Sample Demographics (N = 305 dyads)

Characteristic	Stroke Survivor	Caregiver
	*n* (%)	*n* (%)
Age (years; mean±*SD*)	58.42±11.92	46.08±14.36
Gender		
Male	204 (66.9)	191 (62.6)
Female	101 (33.1)	114 (37.4)
Educational level		
Elementary school	132 (43.3)	66 (21.6)
Junior high school	103 (33.8)	98 (32.1)
Senior high school	53 (17.4)	69 (22.6)
University	17 (5.6)	72 (23.6)
Monthly income (RMB)		
<1,000	24 (7.9)	34 (11.2)
1,000–1,999	79 (25.9)	46 (15.1)
2,000–2,999	104 (34.1)	89 (29.2)
3,000–3,999	62 (20.3)	71 (23.2)
≥4,000	36 (11.8)	65 (21.4)
Occupational status		
Employed	—	164 (53.8)
Departure	—	28 (9.2)
Unemployed/retired	—	113 (37.0)
Understanding of the condition		
Complete	51 (16.7)	56 (18.4)
Partial	226 (74.1)	206 (67.7)
No knowledge at all	28 (9.2)	42-(13.9)
No. strokes		
One	195 (63.9)	—
Two	66 (21.6)	—
Three times and more	44 (14.4)	—
Type of stroke		
Ischemic stroke	202 (66.3)	—
Hemorrhagic stroke	72 (23.6)	—
Mixed stroke	31 (10.1)	—
The frequency of talking to the survivor about their condition		
Frequently	—	106 (34.8)
At times	—	131 (43.0)
Now and then	—	68 (22.3)
Daily care hours		
≤8	—	86 (28.2)
9–11	—	109 (35.7)
≥12	—	110 (36.1)
Relationship with stroke survivor		
Spouse	—	121 (39.7)
Parent	—	3 (0.9)
Child	—	161 (52.8)
Sister/brother	—	20 (6.6)

	Mean ± *SD*	Mean ± *SD*
Health literacy	24.68±4.30	24.42±3.14
Decision self-efficacy	27.97±8.18	29.53±6.62
Decisional conflict	37.26±16.46	38.80±7.41

*Note.* RMB = renminbi.

### Correlations Among Health Literacy, Decision Self-Efficacy, and Decisional Conflict

**Figure 2:**
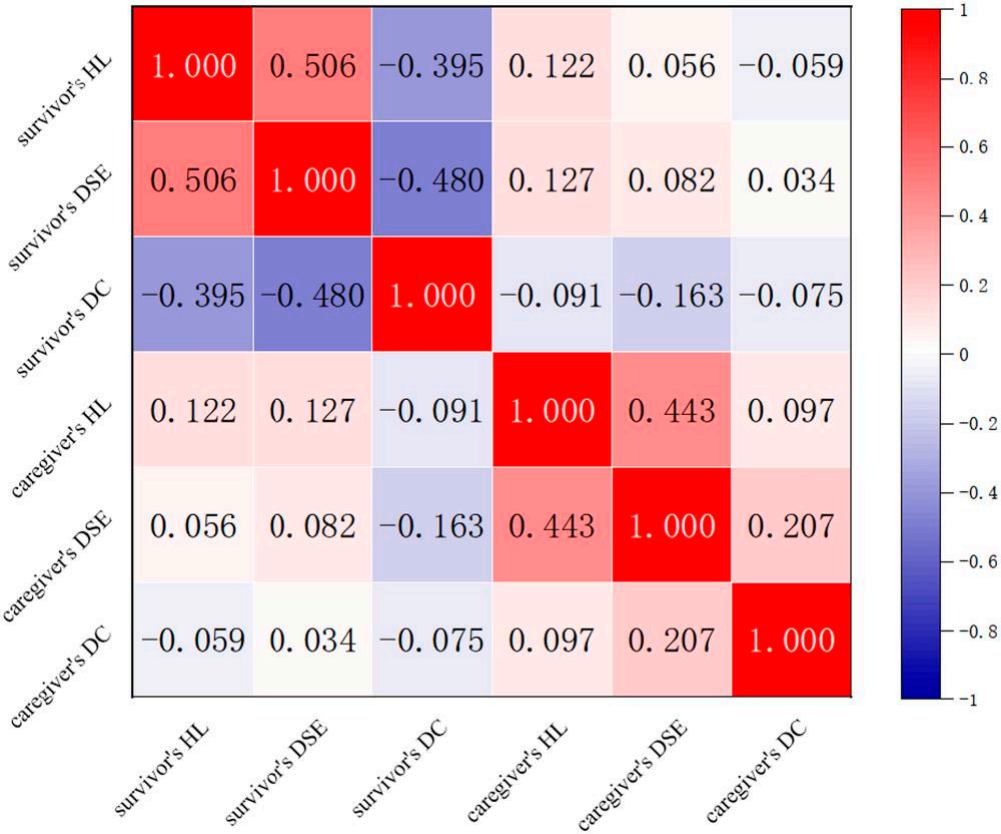
Correlation Heat Map of Dyadic Group Variables *Note.* DC = decisional conflict; DSE = decision self-efficacy; HL = health literacy.

Intercorrelations among the variables in the stroke dyads are shown in Figure 2 (Table 2). Health literacy in both groups (patients and caregivers) was significantly and positively correlated with their own and their caregivers’ decision self-efficacy (*p* < .01). Decisional conflict in patients was shown to correlate significantly and negatively with their own health literacy (*p* < .01) and decision self-efficacy (*p* < .01). However, decisional conflict in the caregivers was shown to correlate significantly and positively with their own decision self-efficacy (*p* < .01).

**Table 2:** Intradyad Correlations Among Health Literacy, Decision Self-Efficacy, and Decisional Conflict

Variable	1	2	3	4	5	6
1. Health literacy ^a^	1					
2. Decision self-efficacy ^a^	.506**	1				
3. Decisional conflict ^a^	−.395**	−.480**	1			
4. Health literacy ^b^	.122*	.127*	−.091*	1		
5. Decision self-efficacy ^b^	.056	.082	−.163**	.443**	1	
6. Decisional conflict ^b^	−.059	.034	−.075	.097	.207**	1

^a^ Stroke survivors. ^b^ caregivers.

*p < .05. **p < .01.

### Actor and Partner Effects

**Figure 3:**
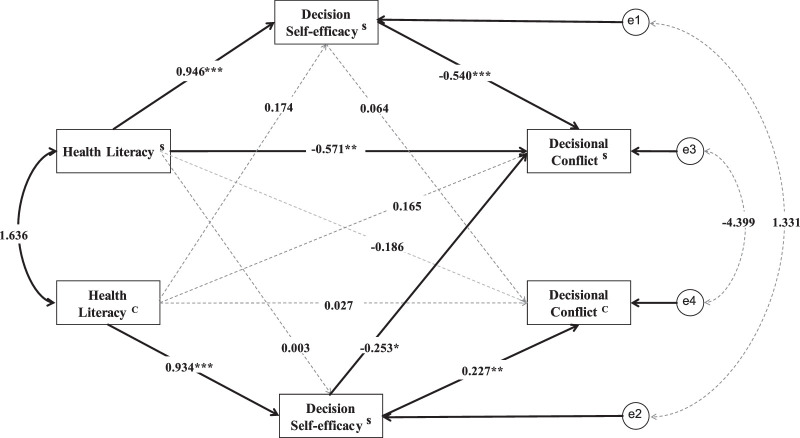
Final Model of Stroke Survivor–Caregiver Health Literacy, Decision Self-Efficacy, and Decisional Conflict *Note.* Solid line indicates significant values. ^S^ Stroke survivors. ^C^ Caregivers. *p < .05. **p < .01. ***p < .001.

The data fit for Model 1 was acceptable (χ^2^/ *df* = 2.754, RMSEA = 0.050, CFI = .998, TLI = .949), with the direct, indirect, and total indirect effects shown in Table 3 and the standard coefficients shown in Figure 3.

**Table 3:** Total, Direct, and Indirect Effects in the APIMeM (N = 305 dyads)

Effect	Estimate	*SE*	95% CI	*p*
**Actor effect**				
Stroke survivors				
Total indirect effect	−0.511	0.128	[−.779, −.283]	**<.001**
HL ^S^→DSE ^S^→DC ^S^	−0.511	0.126	[−.774, −.284]	**<.001**
HL ^S^→DSE ^C^→DC ^S^	−0.001	0.023	[−.050, .044]	.972
Direct effect	−0.571	0.115	[−.947, .190]	**.004**
Caregivers				
Total indirect effect	0.223	0.077	[.067, .376]	**.004**
HL ^C^→DSE ^C^→DC ^C^	0.212	0.077	[.058, .363]	**.006**
HL ^C^→DSE ^S^→DC ^C^	0.011	0.015	[−.010, .050]	.472
Direct effect	0.027	0.418	[−.380, .035]	.858
**Partner effect**				
Stroke survivors				
Total indirect effect	−0.330	0.118	[−.573, −.111]	**.005**
HL ^C^→DSE ^C^→DC ^S^	−0.236	0.101	[−.451, −.059]	**.019**
HL ^C^→DSE ^S^→DC ^S^	−0.094	0.075	[−.253, .043]	.211
Direct effect	0.165	0.237	[−.268, .661]	.485
Caregivers				
Total indirect effect	0.061	0.058	[−.062, .166]	.288
HL ^S^→DSE ^S^→DC ^C^	0.064	0.054	[−.053, .160]	.259
HL ^S^→DSE ^C^→DC ^C^	0.001	0.020	[−.041, .042]	.971
Direct effect	−0.186	0.016	[−.080, .035]	.079

*Note.* APIMeM = Actor–Partner Interdependence Mediation Model; DSE = decision self-efficacy; DC = decisional conflict; HL = health literacy. Bold values indicate statistically significant differences at *p* < .05.

^S^ Stroke survivors. ^C^ Caregiver.

In terms of the direct actor effect, health literacy level in the stroke survivors was found to significantly influence their own decisional conflicts, indicating health literacy relates negatively to decisional conflict in survivors (β = −0.571, *p* = .004). No significant direct actor effect was found for the caregivers.

Mediation analyses were performed using bias-corrected percentile bootstrap (5,000 resamples), with the mediation effect deemed to be insignificant when the Bootstrap 95% CI contained zero. The results revealed a statistically significant indirect actor effect for the stroke survivors, indicating survivors’ decision self-efficacy mediates the relationship between health literacy and decisional conflict (β = −0.511, *p* < .001). Among caregivers, the indirect actor effect was also shown to be statistically significant (β = 0.212; *p* = .006). Among partner effects, only one indirect pathway (caregiver health literacy → caregiver decision self-efficacy → survivor decisional conflict) was identified as significant for survivors (β = −0.236; *p* = .019), suggesting caregiver decision self-efficacy mediates the association between caregiver health literacy and survivor decisional conflict.

## Discussion

To the best of the authors’ knowledge, this was the first study to employ APIMeM to examine the mediating role of decision self-efficacy between health literacy and decisional conflict in the stroke survivor–caregiver dyads, which may enhance scholarly understanding of interaction mechanisms influencing dyad groups. The findings suggest decision self-efficacy partially mediates the association between health literacy and decisional conflict in stroke survivor–caregiver dyads. Health literacy was shown to be associated with decision self-efficacy, which is associated with decisional conflict in stroke survivors and their caregivers. Specifically, better health literacy in caregivers was associated with their having better decision self-efficacy, which was associated with lower decisional conflict in survivors. These findings shed further light on the interactions and interdependence between survivors and caregivers in the decision-making process and may help health care professionals tailor family-centered dichotomous interventions.

In this study, the mean decisional conflict score for caregivers (38.80) was higher than that for stroke survivors (37.26). This contradicts the findings of a previous study (Garvelink et al., 2019) of no significant difference in decisional conflict scores between self-decision-making and others’ proxy decision-making. This discrepancy may be partially explained by cultural differences between China and the West (Gavazzi et al., 2023). Chinese culture places greater emphasis on family responsibility and family closeness. Caregivers are both the main supporters of stroke survivors and family decision-makers. Thus, when making decisions, caregivers not only need to take into account the mood changes and emotional experience of their care recipient (Fadem, 2023) but also consider the expectations of family members as well as their own emotional stress. This double burden may exacerbate the decisional conflict perceived by caregivers. In contrast, in the West, where individualism is more dominant, and decision-making is more personalized, caregivers may experience lower levels of emotional and responsibility stress. Inconsistencies between the participants in different studies may also partially explain the discrepancies found. Whereas Garvelink et al. (2019) used a more balanced sample group covering a wide range of decision-making strategies, the focus of this study was on stroke survivors and their caregivers. Thus, the specificity of the sample may have exposed caregivers to greater emotional burdens and pressures in the decision-making process.

The results of this study show the health literacy of stroke survivors as relating negatively to their own decisional conflicts (i.e., with a significant direct actor effect), which is consistent with the results of previous studies (De Oliveira et al., 2018; Pel-Littel et al., 2024). This suggests patients with higher levels of health literacy are able to effectively access and interpret health information as well as better understand medical information and the risks and benefits of treatment options, which helps reduce confusion and uncertainty in decision-making, improve health intentions, and reduce decisional conflict (Pel-Littel et al., 2024). The results of this study further emphasize the need for using appropriate interventions in treatment and rehabilitation decision-making processes to improve health literacy in patients. However, this study also found that health literacy in caregivers did not directly or significantly influence their own decisional conflicts (i.e., the direct actor effect was not significant), a finding that may be attributed to caregiver emotional and mental vulnerability when faced with decision-making, as described by Miller et al. (2016). Caregivers may exhibit greater emotional volatility and uncertainty in decision-making due to factors such as emotional involvement, stress, and fatigue (Carson et al., 2016; Miller et al., 2016), which may attenuate the positive influence of health literacy on decisional conflict. In light of this, future research should further explore how caregiver decision-making may be improved through psychological support and emotion–management interventions.

In the stroke survivor group, health literacy was found to relate indirectly to their own decisional conflict, which was partially mediated by their own decision self-efficacy. This supports that greater patient knowledge regarding their own health and treatment leads to greater self-efficacy in decision-making (Finset & Street, 2023), thus reducing uncertainty and conflict in the treatment decision-making process. This finding is supported by previous research on colorectal cancer patients (Lee & Bryant-Lukosius, 2023) that found higher informational awareness among patients to be associated with greater decision self-efficacy, a factor that is negatively correlated with decisional conflict. Also, patients with higher decision self-efficacy have been shown to be better able to clearly recognize the advantages and disadvantages of different options and to make the best choices based on their own needs, thus effectively alleviating the uncertainty and anxiety arising from the decision-making process (Yang et al., 2023).

One unexpected result of this study was that enhanced decision self-efficacy among the caregivers was shown to increase their own decisional conflict. This is inconsistent with Lu et al. (2023), which found the level of decisional conflict of decision-making agents to be negatively associated with the level of decision self-efficacy. One potential reason for this discrepancy is differences in information overload between studies. As noted in Parra-Medina and Álvarez-Cervera (2021), information overload depletes limited cognitive resources. In this study, caregivers were faced with long-term home care and rehabilitation exercise decisions involving large numbers of routine care and treatment choices that are information-intensive and complex. While health literacy in caregivers improves self-confidence in certain decision-making contexts, under conditions of information overload (Kim, 2021), caregivers may be unable to critically appraise the information, leading to longer decision-making delays and decision-making conflicts (Davis et al., 2015). Differences in decision-making environments and contexts may be another potential reason for the discrepancy between study findings. In this study, greater attention was paid to the family caregivers of stroke survivors, who are faced with decisions about long-term home care and rehabilitation exercises that involve relatively more information-intensive and complex daily care and treatment choices. Caregivers must not only consider their patients’ current health status but also anticipate and plan for possible future conditions. This complexity and uncertainty may cause caregivers with high self-efficacy to fall into decision-making dilemmas due to overthinking or focusing on the details when confronted with certain decision-making contexts.

In addition, in this study, higher caregiver decision self-efficacy was shown to be associated with less decisional conflict in stroke survivors, while caregiver decision self-efficacy was found to mediate the relationship between caregiver health literacy and patient decisional conflict. This finding underscores the importance of caregivers as critical support in the decision-making processes of stroke survivors. Interdependence theory emphasizes that the attitudes and behaviors of individuals in close relationships influence each other (Wickham & Knee, 2012) and that, in health care decision-making, caregivers are not only the acquirers and managers of information but are also important agents in the decision-making process (Thompson et al., 2020). This has been confirmed in previous research showing that caregivers often face multiple stressors in caring for stroke survivors (Bartoli et al., 2024). When caregivers have high decision self-efficacy, they tend to be more confident in their decision-making abilities and better able to respond positively to the caregiving process, thus reducing their own caregiving burden (Mei et al., 2023). This allows them to reduce decision-making hesitation and conflict due to their own stress when making shared decisions with patients, which in turn reduces patient decision-making conflict. In addition, the shared decision-making model also emphasizes that caregivers not only facilitate the transfer of information but are also active participants in the decision-making process (Faiman & Tariman, 2019). Caregivers with high decision self-efficacy are better able to engage in shared decision-making with their patients, encourage them to express their wishes and preferences, and fully consider their wishes and interests (Mei et al., 2023). This shared decision-making model not only fully utilizes the strengths of both caregiver and patient but also enhances the patient’s sense of identity and satisfaction with their decision and effectively lessens their decisional conflict.

### Limitations

This study was affected by several limitations. First, the cross-sectional research design used cannot capture the dynamic evolution of decisional conflicts over the caregiving process. Future studies may adopt longitudinal designs to track trends at different points in time to gain a deeper understanding of the dyadic decision-making mechanism. Second, this study focused on stroke survivors and their caregivers in China. Future studies may conduct cross-cultural comparisons covering different countries and cultural contexts. Finally, the participants in this study were recruited from six hospitals, which may have introduced selection bias. Future studies may recruit samples from larger, more diverse populations to improve the representativeness/generalizability of the study and its conclusions.

### Conclusions

The findings of this study support that decisional conflict in both treatment and rehabilitation affects stroke survivor–caregiver dyads. Furthermore, caregiver health literacy and decision self-efficacy were both found to have ameliorative effects on decisional conflict in stroke survivors, suggesting patients and caregivers should be viewed as an interdependent and collaborative whole in the stroke care decision-making process. In clinical practice, structured decision-making coaching programs may be implemented to help caregivers systematically acquire the knowledge and skills needed to deal with complex decision-making situations. Second, concise and evidence-based decision-making aids should be developed to improve caregiver efficiency and accuracy in terms of processing critical stroke care–related information and assessing care-related risks. In summary, health literacy education and communication skills training should be combined to enhance caregiver confidence and competence in collaborative decision-making and to minimize intradyad decisional conflicts.

## References

[R1] Andrades-González I. Molina-Mula J. (2022). Validation of content for an app for caregivers of stroke patients through the Delphi method. International Journal of Environmental Research and Public Health, 19(12), Article 7523. 10.3390/ijerph19127523 PMC9223464 35742772

[R2] Aoki Y. Nakayama K. (2023). Improving older adults stroke survivors’ decision-making when selecting a discharge location: A randomized controlled trial protocol. International Journal of Nursing Knowledge, 34(3), 185–192. 10.1111/2047-3095.12393 36114800

[R3] Bandura A. (1977). Self-efficacy: Toward a unifying theory of behavioral change. Psychological Review, 84(2), 191–215. 10.1037//0033-295x.84.2.191 847061

[R4] Bartoli D. Brugnera A. Grego A. Alvaro R. Vellone E. Pucciarelli G. (2024). Stroke disease-specific quality of life trajectories and their associations with caregivers’ anxiety, depression, and burden in stroke population: A longitudinal, multicentre study. European Journal of Cardiovascular Nursing, 23(2), 160–168. 10.1093/eurjcn/zvad054 37249041

[R5] Berkman N. D. Sheridan S. L. Donahue K. E. Halpern D. J. Crotty K. (2011). Low health literacy and health outcomes: An updated systematic review. Annals of Internal Medicine, 155(2), 97–107. 10.7326/0003-4819-155-2-201107190-00005 21768583

[R6] Bunn H. O’Connor A. (1996). Validation of client decision-making instruments in the context of psychiatry. Canadian Journal of Nursing Research, 28(3), 13–27. 8997937

[R7] Carson S. S. Cox C. E. Wallenstein S. Hanson L. C. Danis M. Tulsky J. A. Chai E. Nelson J. E. (2016). Effect of palliative care-led meetings for families of patients with chronic critical illness: A randomized clinical trial. JAMA-Journal of the American Medical Association, 316(1), 51–62. 10.1001/jama.2016.8474 PMC5538801 27380343

[R8] Chinn D. McCarthy C. (2013). All Aspects of Health Literacy Scale (AAHLS): Developing a tool to measure functional, communicative and critical health literacy in primary healthcare settings. Patient Education and Counseling, 90(2), 247–253. 10.1016/j.pec.2012.10.019 23206659

[R9] Comulada W. S. (2021). Calculating level-specific SEM fit indices for multilevel mediation analyses. Stata Journal, 21(1), 195–205. 10.1177/1536867x211000022 33935596 PMC8087284

[R10] Davis E. L. McCaffery K. Mullan B. Juraskova I. (2015). An exploration of decision aid effectiveness: The impact of promoting affective vs. deliberative processing on a health-related decision. Health Expectations, 18(6), 2742–2752. 10.1111/hex.12248 25228065 PMC5810680

[R11] De Oliveira G. J. Errea M. Bialek J. Kendall M. C. McCarthy R. J. (2018). The impact of health literacy on shared decision making before elective surgery: A propensity matched case control analysis. BMC Health Services Research, 18(1), Article 958. 10.1186/s12913-018-3755-9 PMC6292056 30541541

[R12] Fadem S. (2023). Investigating and supporting patient and caregiver sensemaking in complex medical decisions using participatory design. MDM Policy & Practice, 8(1), 1–11. 10.1177/23814683231164988 PMC10107376 37077898

[R13] Faiman B. Tariman J. D. (2019). Shared decision making: Improving patient outcomes by understanding the benefits of and barriers to effective communication. Clinical Journal of Oncology Nursing, 23(5), 540–542. 10.1188/19.CJON.540-542 31538972

[R14] Finset A. Street R. J. (2023). Risk communication, shared decision making and health literacy. Patient Education and Counseling, 116, Article 107983. 10.1016/j.pec.2023.107983 37741230

[R15] Garvelink M. M. Boland L. Klein K. Nguyen D. V. Menear M. Bekker H. L. Eden K. B. LeBlanc A. O’Connor A. M. Stacey D. Légaré F. (2019). Decisional conflict scale findings among patients and surrogates making health decisions: Part II of an anniversary review. Medical Decision Making, 39(4), 315–326. 10.1177/0272989X19851346 31142205

[R16] Gavazzi G. Noferini C. Benedetti V. Cotugno M. Giovannelli F. Caldara R. Mascalchi M. Viggiano M. P. (2023). Cultural differences in inhibitory control: An ALE meta-analysis. Brain Sciences, 13(6), Article 907. 10.3390/brainsci13060907 PMC10295933 37371385

[R17] Glick D. R. Motta M. Wiegand D. L. Range P. Reed R. M. Verceles A. C. Shah N. G. Netzer G. (2018). Anticipatory grief and impaired problem solving among surrogate decision makers of critically ill patients: A cross-sectional study. Intensive and Critical Care Nursing, 49, 1–5. 10.1016/j.iccn.2018.07.006 30057337

[R18] Goh Z. Chia J. Seow T. Y. Choo J. Foo M. Seow P. S. Griva K. (2022). Treatment-related decisional conflict in pre-dialysis chronic kidney disease patients in Singapore: Prevalence and determinants. British Journal of Health Psychology, 27(3), 844–860. 10.1111/bjhp.12577 34865298

[R19] Guo Z. Zeng S. Ling K. Chen S. Yao T. Li H. Xu L. Zhu X. (2024). Experiences and needs of older patients with stroke in China involved in rehabilitation decision-making: A qualitative study. BMC Medical Informatics and Decision Making, 24(1), Article 330. 10.1186/s12911-024-02735-5 PMC11539789 39506728

[R20] Hilkens N. A. Casolla B. Leung T. W. de Leeuw F. E. (2024). Stroke. Lancet, 403(10446), 2820–2836. 10.1016/S0140-6736(24)00642-1 38759664

[R21] Kim S. (2021). Caregivers’ information overload and their personal health literacy. Western Journal of Nursing Research, 43(5), 431–441. 10.1177/0193945920959086 32948106

[R22] Ledermann T. Macho S. (2009). Mediation in dyadic data at the level of the dyads: A Structural Equation Modeling approach. Journal of Family Psychology, 23(5), 661–670. 10.1037/a0016197 19803602

[R23] Lee M. K. Bryant-Lukosius D. (2023). Information provision, decision self-efficacy, and decisional conflict in adopting health behaviors among patients treated for colorectal cancer: A cross-sectional study. Cancer Nursing, 46(1), 45–56. 10.1097/NCC.0000000000001040 34817417

[R24] Lu S.-J. Ku S.-C. Liu K.-F. Chien C.-H. (2023). Decision self-efficacy and decisional conflict on reintubation among surrogates of ventilated patients undergoing planned extubation. Asian Nursing Research, 17(5), 235–244. 10.1016/j.anr.2023.10.001 37838098

[R25] Lynn C. Quast L. Rogers H. Effinger K. Gilleland-Marchak J. (2020). Systematic review of health literacy in childhood cancer patients, survivors, and their caregivers. Journal of Pediatric Psychology, 45(4), 373–385. 10.1093/jpepsy/jsaa009 32186708

[R26] Martin S. S. Aday A. W. Almarzooq Z. I. Anderson C. Arora P. Avery C. L. Baker-Smith C. M. Barone G. B. Beaton A. Z. Boehme A. K. Commodore-Mensah Y. Currie M. E. Elkind M. Evenson K. R. Generoso G. Heard D. G. Hiremath S. Johansen M. C. Kalani R., … Palaniappan L. P. (2024). 2024 heart disease and stroke statistics: A report of US and global data from the American Heart Association. Circulation, 149(8), e347–e913. 10.1161/CIR.0000000000001209 38264914 PMC12146881

[R45] Marziliano A. Miller S. M. Fleisher L. G. Ropka M. E. Stanton A. L. Wen K. -Y. Cornelius T. Lapitan E. Diefenbach M. A. (2023). Examining the impact of a multimedia intervention on decisional conflict and psychological distress among early-stage breast cancer patients: Results from a nationwide RCT. Translational Behavioral Medicine, 13(10), 727–735. 10.1093/tbm/ibad037 37379519 PMC10538468

[R27] Mei Y. X. Xiang D. D. Zhang Z. X. Twumwaah B. J. Lin B. L. Chen S. Y. (2023). Family function, self-efficacy, care hours per day, closeness and benefit finding among stroke caregivers in China: A moderated mediation model. Journal of Clinical Nursing, 32(3-4), 506–516. 10.1111/jocn.16290 35285125

[R28] Miller J. J. Morris P. Files D. C. Gower E. Young M. (2016). Decision conflict and regret among surrogate decision makers in the medical intensive care unit. Journal of Critical Care, 32, 79–84. 10.1016/j.jcrc.2015.11.023 26810482

[R29] Muscat D. M. Shepherd H. L. Nutbeam D. Trevena L. McCaffery K. J. (2021). Health literacy and shared decision-making: Exploring the relationship to enable meaningful patient engagement in healthcare. Journal of General Internal Medicine, 36(2), 521–524. 10.1007/s11606-020-05912-0 32472490 PMC7878628

[R30] O’Connor A. M. (1995). Validation of a decisional conflict scale. Medical Decision Making, 15(1), 25–30. 10.1177/0272989X9501500105 7898294

[R31] Parra-Medina L. E. Álvarez-Cervera F. J. (2021). Information overload syndrome: A bibliographic review. Revista De Neurologia, 73(12), 421–428. 10.33588/rn.7312.2021113 34877645

[R32] Pel-Littel R. E. Buurman B. M. Minkman M. M. Scholte O. R. W. Twisk J. van Weert J. (2024). The influence of health literacy, anxiety and education on shared decision making and decisional conflict in older adults, and the mediating role of patient participation: A video observational study. Patient Education and Counseling, 124, Article 108274. 10.1016/j.pec.2024.108274 38547640

[R33] Pu L. Wang L. Zhang R. Zhao T. Jiang Y. Han L. (2023). Projected global trends in ischemic stroke incidence, deaths and disability-adjusted life years from 2020 to 2030. Stroke, 54(5), 1330–1339. 10.1161/STROKEAHA.122.040073 37094034

[R34] Raykov T. Tomer A. Nesselroade J. R. (1991). Reporting structural equation modeling results in Psychology and Aging: Some proposed guidelines. Psychology and Aging, 6(4), 499–503. 10.1037//0882-7974.6.4.499 1777136

[R35] Roshan A. G. Hosseinkhani S. N. Norouzadeh R. (2023). Health literacy and self-efficacy of the elderly with diabetes. Journal of Diabetes and Metabolic Disorders, 22(1), 611–617. 10.1007/s40200-023-01181-w 37255792 PMC10225399

[R36] Thompson C. J. Bridier N. Leonard L. Morse S. (2020). Exploring stress, coping, and decision-making considerations of Alzheimer’s family caregivers. Dementia-International Journal of Social Research and Practice, 19(6), 1907–1926. 10.1177/1471301218809865 30486660

[R37] van Lent L. de Jonge M. van der Ham M. van Mil M. Gort E. H. Hasselaar J. Oomen-de H. E. van der Rijt C. van Weert J. Lolkema M. P. (2022). Decisional conflict after deciding on potential participation in early phase clinical cancer trials: dependent on global health status, satisfaction with communication, and timing. Cancers, 14(6), Article 1500. 10.3390/cancers14061500 PMC8946532 35326653

[R38] Wang L. Chen Y. Cui J. Fang H. Liu H. Liao Z. Hu K. (2019). The reliability and validity test of the chinese version of the decision conflict scale in the surgical decision-making of rectal cancer patients. Journal of Nursing, 34(3), 31–35. (Original work published in Chinese)

[R39] Wang S. Ye Z. Li Y. Liu F. Li L. (2021). The reliability and validity test of the chinese version of the decision self-efficacy scale in the treatment decision-making of patients with primary liver cancer. Journal of Nursing of the People’s Liberation Army, 38(1), 37–40.

[R40] Wickham R. E. Knee C. R. (2012). Interdependence theory and the actor-partner interdependence model: Where theory and method converge. Personality and Social Psychology Review, 16(4), 375–393. 10.1177/1088868312447897 22619276

[R41] Wu Q. Ye X. Wu Y. Zhao L. Wu J. (2017). The localization and reliability and validity analysis of the comprehensive health literacy measurement scale. Chinese General Practice, 20(10), 1229–1233.

[R42] Yang S. Yu L. Zhang C. Xu M. Tian Q. Cui X. Liu Y. Yu S. Cao M. Zhang W. (2023). Effects of decision aids on breast reconstruction: A systematic review and meta-analysis of randomised controlled trials. Journal of Clinical Nursing, 32(7-8), 1025–1044. 10.1111/jocn.16328 35460127

[R43] Yuen E. Wilson C. Livingston P. M. White V. McLeod V. Dufton P. H. Hutchinson A. M. (2023). Caregiver and care recipient health literacy, social support and connectedness on caregiver psychological morbidity: A cross-sectional dyad survey. Psycho-Oncology, 32(8), 1257–1267. 10.1002/pon.6177 37430441

[R44] Zhang X. Jiang L. Yu Y. (2021). A qualitative study on the participation of patients with acute ischemic Stroke and their families in intravenous thrombolysis decision-making. Journal of Nantong University, 41(5), 463–466. 10.16424/j.cnki.cn32-1807/r.2021.05.018

